# Efficacy and Safety of Indigo Naturalis in Combination with Narrow-Band Ultraviolet B for Treatment of Pityriasis Rosea: A Meta-Analysis

**DOI:** 10.1155/2018/6816981

**Published:** 2018-03-27

**Authors:** L. Wang, Y. N. Xue, Z. W. Li, W. Zhang, X. P. Ji, Z. Fan, Y. J. Li

**Affiliations:** ^1^Department of Scientific Research, The First Affiliated Hospital, Xi'an Medical University, Xi'an, China; ^2^Department of Pharmacy, The Second Hospital of Hanbin, Ankang, China; ^3^Department of Traditional Chinese Medicine, The First Affiliated Hospital, Xi'an Medical University, Xi'an, China; ^4^Department of Dermatology, The First Affiliated Hospital, Xi'an Medical University, Xi'an, China

## Abstract

Pityriasis rosea (PR), a skin rash, causes substantial discomfort in patients. There is a lack of effective therapies for PR. A combination of ultraviolet irradiation and* indigo naturalis* treatment has been shown to be a safe and effective regime for control of PR; however, the data have been largely inconsistent. Tis meta-analysis further evaluated the efficacy and safety of this combination in patients with PR. The PubMed, Embase, Cochrane Library, CNKI, VIP, and Wan Fang databases were searched for relevant RCTs of this combination therapy in patients with PR. A total of eight studies with a combined study population of 688 patients published between January 2006 and March 2016 were eligible for this meta-analysis. The RevMan 5.3 software was used for the meta-analysis. The regimen of compound* indigo naturalis* plus NB-UVB showed much better control of PR as compared to that achieved with use of compound* indigo naturalis* or NB-UVB alone in terms of cure rate or effective rate. However, no significant difference was observed between the two with respect to incidence of adverse effects. The analysis was affected by publication bias as revealed by funnel plot analysis. Further studies with large sample sizes are required to confirm our findings.

## 1. Introduction

Pityriasis rosea (PR) is a common skin disorder characterized by rash and substantial discomfort. Clinically, PR is an acute self-limiting inflammatory disease that accounts for 0.3%–3% of new patient consultations at dermatology clinics [1]. PR more commonly affects children and young adults; peak incidence is in the age group of 10–35 years. The disease is uncommon in patients younger than 10 years of age [2]. The disease typically resolves within one to three months; however, it significantly impacts the quality of life of the patients. The exact cause of PR is not clear; however, it may be related to viral infection, especially infection with human herpes viruses 6 and 7 [3, 4]. Antiviral therapy (such as acyclovir) has been shown to reduce the duration and severity of the disease [5–7]. The diagnosis of PR is usually based on clinical signs and symptoms. A set of validated diagnostic criteria for PR are available; these include essential clinical features of discrete circular or oval lesions, scaling on most lesions, or peripheral collarette scaling with central clearance in at least two lesions [8, 9]. There are several forms of PR including the relapsing form, the persistent form, and the pityriasis rosea-like eruptions [10–13]. Treatment of PR is usually symptomatic and there is no evidence to support the efficacy of specific therapies. However, previous studies have shown that ultraviolet irradiation and/or indigo naturalis are safe and effective treatment options for control of PR. Exposure to direct sunlight was shown to induce quick resolution of the lesions, which is the underlying rationale for the use of ultraviolet irradiation as a treatment modality [14].

*Indigo naturalis* (Qing Dai), extracted from the stems and leaves of* Baphicacanthus cusia *(Ness) Bremek, has been commonly used as a traditional Chinese remedy for various inflammatory disorders including dermatoses [15]. Oral administration of* indigo naturalis* has been used to treat psoriasis in China and its efficacy has been proved in several clinical studies [16]. Irradiation with narrow-band ultraviolet B (NB-UVB) at 311 nm wavelength was shown to alleviate erythema associated with lesions or rash [17], inhibiting keratinocyte proliferation. Oral compound indigo naturalis capsule alleviates “pathogenic heat” (a traditional Chinese medicine term, which could be referred to as antiviral effects) from the blood and ecchymosis [17]. Such an external treatment in combination with internal treatment could accrue full advantage of Chinese and Western medicine to effectively control PR and shorten the disease course. However, the efficacy of such combination treatment is still unclear or inconsistent. Approximately 50% of patients with PR experience moderate-to-severe itch and it is not known whether such treatment is effective and whether the combination could outweigh the risk of adverse effects. Thus, in this meta-analysis, we further evaluated the efficacy and safety of this combination in patients with PR.

## 2. Materials and Methods

### 2.1. Data Sources and Search Strategy

We conducted a search for randomized controlled trials of compound indigo naturalis in combination with NB-UVB in patients with pityriasis rosea on PubMed, Embase, the Cochrane Library, the China National Knowledge Infrastructure (CNKI) database, and the Chinese Scientific Journals Full-Text Database (CQVIP). The reference period for the literature search was from January 2006 to March 2016. We utilized the search terms of compound indigo naturalis, narrow-band ultraviolet B, rose rosea, and randomized controlled trials with adjustments for different databases. The reference lists of eligible studies retrieved from the databases were manually searched to identify additional studies. During extraction of publications, we avoided subjective bias by omitting names of the authors, journals, year, and country.

### 2.2. Inclusion and Exclusion Criteria

The abstracts of the retrieved studies were independently searched and reviewed by two investigators (W. Zhang and Z. W. Li) and the full texts of the selected studies were further evaluated for eligibility and inclusion in the meta-analysis. Any disagreements were resolved by consensus after consultation with a third reviewer (Z. Fan). A study was considered eligible if it met the following criteria: (1) patients with rose rosea diagnostic criteria; (2) patients did not receive corticosteroids, immunosuppressants, antihistamines, or other drugs in 2 weeks; and (3) patients without other diseases. The exclusion criteria were as follows: (1) patients allergic to UV irradiation; (2) pregnant and lactating women; (3) patients with severe heart, liver, kidney, gastrointestinal, and other diseases; and (4) rose rosea with infection.

### 2.3. Data Extraction

According to Cochrane methodology, two investigators (Z. W. Li and Y. N. Xue) independently extracted data pertaining to study characteristics in a standardized spreadsheet and assessed the methodological quality of the included studies.

### 2.4. Quality Assessment

The quality of this meta-analysis was ensured by scores and all the included studies were categorized according to the Quality Assessment of Diagnostic Accuracy Studies [18], which includes 14 items to assess all included studies.

### 2.5. Interventions and Outcome of Treatment

The treatment group received oral compound indigo pills or capsules in combination with NB-UVB, while the control group received either oral compound indigo pills/capsules or NB-UVB treatment.

The cure rate, the effective rate, and the incidence rate of adverse reactions were assessed in each patient after treatment. Clinical efficacy was rated as follows: (1) cure: rash reduced by >90% and complete resolution of itching; (2) markedly effective: rash reduced by >60% and significant reduction in itching; (3) effective: rash reduced by >30% and reduction in itching; (4) ineffective: rash reduced by 30% or less and no relief of itching.

(1) Recovery  rate%=number  of  cases  curedobserved  number  of  cases×100;Efficiency  rate%=cured  cases+markedly  caseobserved  cases×100.

### 2.6. Statistical Analysis

RevMan 5.3 software [Review Manager version 5.3, Copenhagen: The Nordic Cochrane Centre, The Cochrane Collaboration, 2014] was used to analyze the data, that is, classification of data and calculation of risk ratios (RR) and 95% confidence intervals (95% CI). Heterogeneity across studies was assessed using the Cochrane *I*^2^ statistics. *I*^2^ < 50% was considered indicative of a lack of significant heterogeneity among the included studies and a fixed-effects model was used for analysis. *I*^2^ > 50% was considered indicative of substantial heterogeneity and a random-effects model was used for analysis. In order to assess the influence of individual studies on the pooled analysis, a sensitivity analysis was performed by exclusion of individual studies, one at a time, to determine the stability of the meta-analysis. The analytic results were further shown using the forest plot, while a funnel plot was used to evaluate possible publication bias.

The Ethics Committee of the First Affiliated Hospital of Xian Medical University approved the study.

## 3. Results

### 3.1. Data Retrieval

**Figure 1:**
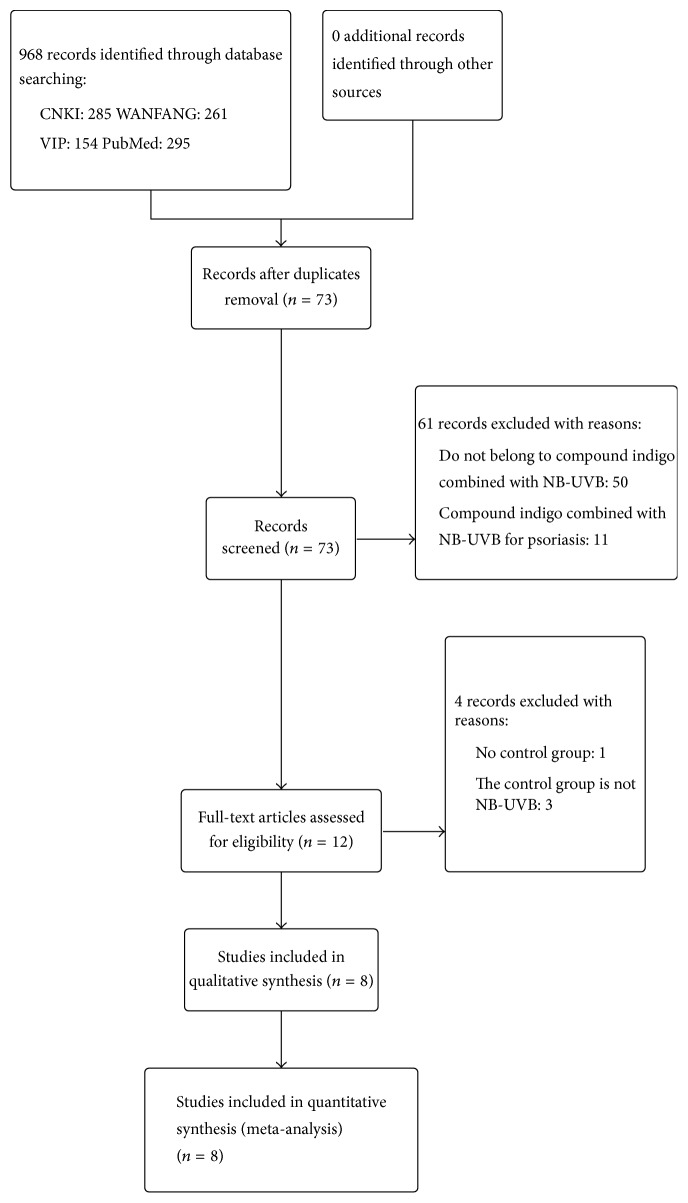
Schematic illustration of the literature search and study selection criteria.

A total of 968 publications were retrieved on initial database search. The titles and abstracts were screened against the inclusion and exclusion criteria. Finally, we obtained eight randomized controlled trials with a combined study population of 688 subjects; among these, 333 subjects were in the treatment group and 355 subjects were in the control group [17, 19–25]. A schematic illustration of the literature search is shown as a flow chart in Figure 1. All eight studies were conducted in China and published in Chinese language.

The basic characteristics of the included studies are listed in Table 1. The age of participants in the included studies ranged between 16 and 85 years. All eight studies specified the cure rate and effective rate, while seven studies specified the adverse reaction that mainly included erythema, thermalgia, itch, and diarrhea (Table 2). Five studies compared combined therapy with compound indigo naturalis, and four studies used NB-UVB as the control intervention. Treatment duration ranged between 2 weeks (3 studies) and 4 weeks (2 studies). Outcome measures included cure rate (8 studies), effective rate (8 studies), and incidence rate of adverse reactions (7 studies).

### 3.2. Data Analysis

#### 3.2.1. Compound Indigo Naturalis in Combination with NB-UVB versus Compound Indigo Naturalis Alone

**Figure 2:**
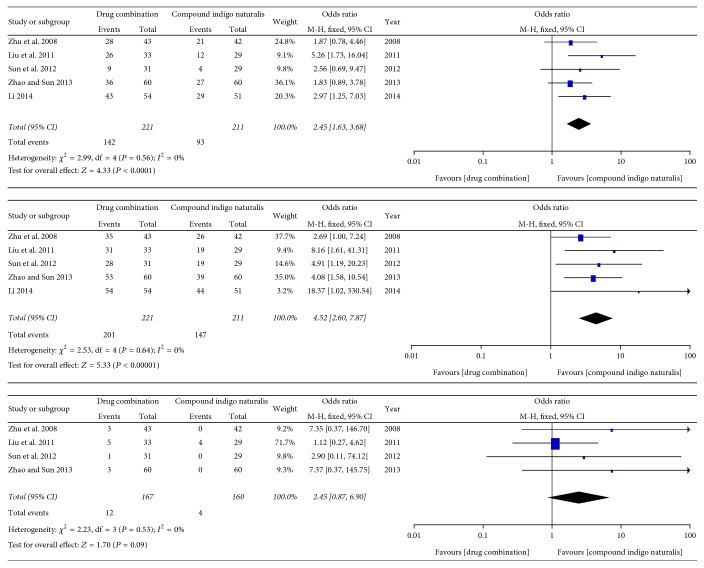
Forest plot analysis of the cure rate, the effective rate, and the incidence rate of adverse reactions associated with treatment of pityriasis rosea with compound indigo naturalis plus NB-UVB versus compound indigo naturalis alone (control).

Five trials reported the cure rate and the effective rate of compound indigo naturalis in combination with NB-UVB and compound indigo naturalis alone as the control intervention. As shown in Figure 2, heterogeneity analysis indicated no significant heterogeneity between these two treatment groups. Therefore, the fixed-effects model was used for data analysis. The data showed that the cure rate and the effective rate of compound indigo naturalis in combination with NB-UVB were higher than that of compound indigo naturalis alone (OR = 2.45, 95% CI: 1.63–3.68, and *P* < 0.0001 and OR = 4.52, 95% CI: 2.60–7.87, and *P* = 0.0001, resp.).

The incidence rates of adverse reactions were reported for four trials (compound indigo naturalis plus NB-UVB versus compound indigo naturalis alone). As shown in Figure 2, no significant heterogeneity was observed between these two groups (*I*^2^ = 0%; *P* = 0.53); therefore, the fixed-effects model was used for data analysis. No significant difference with respect to the incidence of adverse reactions was observed between the experimental and control groups (OR = 2.45, 95% CI: 0.87–6.90, and *P* = 0.09).

#### 3.2.2. Compound Indigo Naturalis in Combination with NB-UVB versus NB-UVB Alone

**Figure 3:**
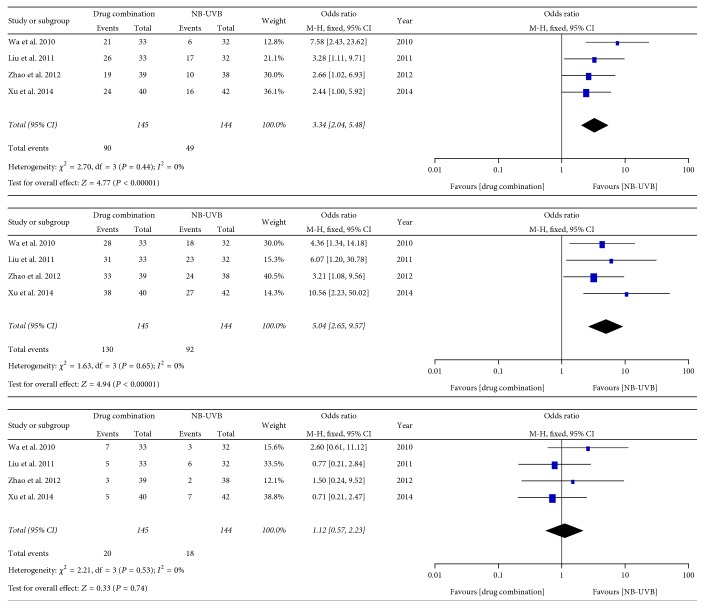
Forest plot analysis of the cure rate, the effective rate, and the incidence rate of adverse reactions associated with treatment of pityriasis rosea with compound indigo naturalis plus NB-UVB versus NB-UVB alone (control).

Five trials reported the cure rate, the effective rate, and the incidence rate of adverse reaction after treatment of pityriasis rosea with compound indigo naturalis plus NB-UVB and NB-UVB alone. As shown in Figure 3, heterogeneity analysis indicated no significant heterogeneity between these two groups and the fixed-effects model was used for data analysis. We found that the cure rate and the effective rate of the combination therapy was much higher than that of NB-UVB alone (the between-group difference was markedly significant (OR = 3.34, 95% CI: 2.04–5.48, and *P* < 0.00001 and OR = 5.04, 95% CI: 2.65–9.57, and *P* < 0.00001, resp.). No significant between-group difference was observed with respect to the incidence rate of adverse reactions (OR = 1.12, 95% Cl: 0.57–2.23, and *P* = 0.74).

#### 3.2.3. Assessment of Publication Bias

**Figure 4:**
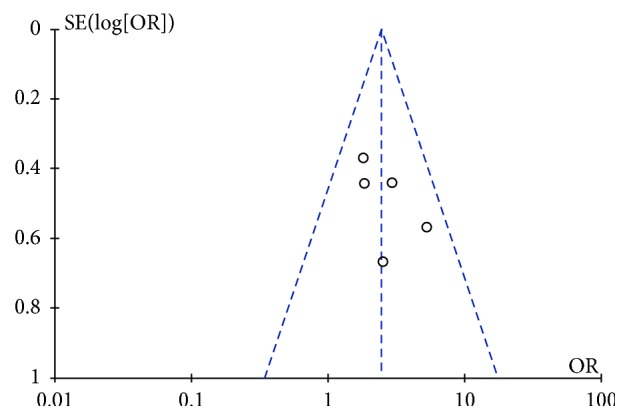
Funnel graph analysis of the cure rate associated with treatment of pityriasis rosea with the compound indigo naturalis plus NB-UVB versus the compound indigo naturalis (control).

Next, we assessed the publication bias using Deeks' test and the funnel plot shows the standard deviation of the effect size as the vertical coordinate and the numerical value as the horizontal coordinate by the effect of each research OR (Figure 4). The funnel plot showed an asymmetrical distribution of the samples which suggests that some of the test methodologies may have been of low quality and that the results may have been influenced by publication bias.

## 4. Discussion

Pityriasis rosea is a commonly encountered inflammatory skin disease in clinical settings. The skin rash typically appears on the trunk and proximal extremities and commonly occurs in the Spring and Autumn seasons. Although the disease is self-limiting [1], the disease course typically lasts for 4 to 8 weeks; recurrent skin rashes may occur in some cases and the disease course may extend from more than half a year to several years. The pathogenesis of pityriasis rosea is not completely understood; viral infection-related immune response and other factors are believed to be involved. In traditional Chinese medicine, such a disease belongs to “pathology heat” and is attributable to “heat” blood accumulation in the skin and prolonged exposure to external factors leading to skin rash. In this regard, indigo naturalis possesses anti-“heat” (antiviral and anti-inflammatory) effects in vitro and has been used for treatment of psoriasis and eczema in clinical settings [26]. The compound indigo capsule is made up of several traditional Chinese medicine herbs, such as indigo,* Salvia miltiorrhiza*, tuckahoe, and shikonin. It inhibits mitosis of the epithelial cells, promotes blood circulation, reduces blood viscosity, and has anti-inflammatory effects [26]. Thus, a combination of NB-UVB plus indigo naturalis could effectively control pityriasis rosea. In the current study, we performed a meta-analysis to evaluate the outcomes of combination regime versus each of the monotherapies in patients with pityriasis rosea. We found that the efficacy of the combination of compound indigo naturalis and NB-UVB was much better than that of compound indigo naturalis or NB-UVB alone in terms of the cure rate and the effective rate. However, the adverse effect rates were comparable between the individual therapies and the combination therapy. Further studies with larger sample sizes will confirm the efficacy of combination regimen for control of pityriasis rosea.

Routine therapies for pityriasis rosea include oral antihistamines, corticosteroids, and antiviral agents in addition to topical use of calamine lotion. However, these therapies often fail to achieve satisfactory disease control. Aberrant immune response is believed to be involved in the pathogenesis of PR; therefore, modulation of immune response is a key therapeutic target in these patients. Although the efficacy of UVB phototherapy has been contested [5–7, 27], NB-UVB with a wavelength of 311–313 nanometers has been found to be the safest treatment for different skin disorders [28]. NB-UVB treatment has been shown to suppress the immune system and attenuate inflammatory response in the skin [28]. Moreover, NB-UVB has strong penetrability and does not cause skin burn or induce an erythematous reaction. Moreover, it does not overlap the DNA absorption peak and is not liable to cause mutation of genomic DNA in the skin cells [17]. NB-UVB treatment considerably reduces the severity of pityriasis rosea and hastens recovery. However, NB-UVB monotherapy for pityriasis rosea does have some limitations; for example, prolonged use of ultraviolet light can cause progressive damage to human skin, such as induction of genomic DNA mutations, damage to skin cells, collagen, and destruction of vitamins A and C in the skin. Thus, combination therapies reduce such side effects of NB-UVB. The present meta-analysis further indicates the superiority of combination regime of compound indigo naturalis plus NB-UVB over monotherapy for control of PR. The side effects of such regime were similar to those of monotherapies. In China, such a regime is called the combination of traditional Chinese medicine with modern Western medicine to achieve maximal effects with a concomitant reduction in side effects of single drug treatment. This synergistic effect may be attributable to improved local blood circulation because of natural medical herbs, which decreases or eliminates the side effects of local treatment, like NB-UVB.

However, our current study does have some limitations. For example, the number of studies and the sample size used in this analysis were relatively small. Moreover, the included studies did show some variability with respect to data analyses. Our results may also have been affected by publication bias. Thus, further validation study with a large cohort and independent patients is needed to confirm our findings. Future studies should investigate the efficacy of this regime with respect to control of erythema, itching, and time required for relief of itch.

## 5. Conclusion

Results from this study demonstrate that combination therapy with compound indigo naturalis plus NB-UVB is superior to monotherapy for control of pityriasis rosea. The side effects of such combination were similar to those of single treatment. Further clinical studies are required to confirm our findings.

## Figures and Tables

**Table 1:** Characteristics of the studies included in the meta-analysis.

Author, year	cases		Intervention and measures		Outcomes
	Combination	Single	Combination	Single	
Zhu et al., 2008 [17]	43	42	Compound indigo capsules p.o. 4 tables t.i.d.; dexamethasone (DXM) cream ad us. exts.l.d NB-UVB irradiation (311 nm): starting irradiation dose 0.39–0.50 J/cm^2^, increased by 0.1 J/cm^2^ every time q.o.d.	Compound indigo capsules p.o. 4 tables t.i.d. Dexamethasone (DXM) cream ad us.exts.l.d	①②③

Wa et al., 2010 [19]	33	32	Compound indigo capsules p.o. 4 tables t.i.d. NB-UVB irradiation: starting irradiation dose 0.5–0.7 MED increased 0.1 J/cm^2^ every time q.o.d.	NB-UVB irradiation: starting irradiation dose 0.5–0.7 MED increased by 0.1–0.2 J/cm^2^ once q.o.d.	①②③

Liu et al., 2011 [20]	33	29/32	Compound indigo capsules p.o. 4 tables t.i.d. NB-UVB irradiation (311–313 nm): starting irradiation dose 0.3–0.5 J/cm^2^ increased by 10%–20% once q.o.d.	Control group 1: compound indigo capsules p.o. 4 tables t.i.d. Control group 2: NB-UVB irradiation (311–313 nm): starting irradiation dose 0.3–0.5 J/cm^2^ increased by 10%–20% once q.o.d.	①②③

Sun et al., 2012 [21]	31	29	Compound indigo pill p.o. 2 g t.i.d. NB-UVB irradiation (311 nm): starting irradiation dose 0.35 J q.o.d. increased by 0.1 J once q.o.d.	Compound indigo pill p.o. 2 g t.i.d.	①③

Zhao et al., 2012 [22]	39	38	Compound indigo pill p.o. 2 g t.i.d. NB-UVB irradiation q.o.d., Calamine lotion ad us.ext	NB-UVB irradiation q.o.d., Calamine lotion ad us.ext	①②③

Zhao and Sun, 2013 [23]	60	60	Compound indigo capsules p.o. 4 tables t.i.d. NB-UVB irradiation exposure done 0.2 J/cm^2^ t.i.w.	Compound indigo capsules p.o. 4 tables t.i.d.	①③

Xu et al., 2014 [24]	40	42	Compound indigo capsules p.o. 4 tables t.i.d. NB-UVB irradiation (311 nm): starting irradiation dose 0.4–0.6 J/cm^2^ b.i.w.	NB-UVB irradiation (311 nm): starting irradiation dose 0.4–0.6 J/cm^2^ b.i.w.	①②③

Li, 2014 [25]	54	51	Compound indigo pills p.o. 6 g t.i.d. NB-UVB irradiation (311 nm): starting irradiation dose 0.4–0.5 J/cm^2^ q.o.d. increased Clobetasol propionate ointment	Compound indigo pills p.o. 6 g t.i.d. Clobetasol propionate ointment	②③

Primary outcome: ① the cure rate; ② the effective rate; and ③ the incidence rate of adverse reaction.

**Table 2:** Adverse reactions in each study.

Included studies	Test group					Control group				
	Adverse reaction	Erythema	Thermalgia	Itch	diarrhea	Adverse reaction	Erythema	Thermalgia	Itch	diarrhea
Zhu et al. 2008 [17]	3	3	3	3	0	0	NA	NA	NA	0
Wa et al. 2010 [19]	7	3	3	NA	4	3	3	3	NA	NA
Liu et al. 2011 [20]	5	5	NA	NA	NA	4	NA	NA	2	NA
Liu et al. 2011 [20]	5	5	NA	NA	NA	6	5	1	NA	NA
Sun et al. 2012 [21]	1	1	NA	NA	NA	0	NA	NA	NA	NA
Zhao et al. 2012 [22]	3	NA	NA	NA	2	2	2	NA	NA	NA
Zhao and Sun 2013 [23]	3	3	NA	NA	NA	8	8	NA	5	NA
Xu et al. 2014 [24]	5	4	1	NA	NA	7	5	2	NA	NA
Li 2014 [25]	3	3	3	NA	NA	NA	NA	NA	NA	NA
